# Evolution of Oxygen Content of Graphene Oxide for Humidity Sensing

**DOI:** 10.3390/molecules29163741

**Published:** 2024-08-07

**Authors:** Xue Zhang, Guocheng Zhang, FuKe Wang, Hong Chi

**Affiliations:** 1 Engineering and Technology Center of Electrochemistry, School of Chemistry and Chemical Engineering, Qilu University of Technology (Shandong Academy of Sciences), Jinan 250353, China; 2 Institute of Materials Research and Engineering (IMRE), Agency for Science, Technology and Research (A*STAR), 2 Fusionopolis Way, Innovis #08-03, Singapore 138634, Singapore

**Keywords:** oxygen-containing functional groups, graphene, humidity, sensitivity

## Abstract

Graphene oxide (GO) has shown significant potential in humidity sensing. It is well accepted that the oxygen-containing functional groups in GO significantly influence its humidity sensing performance. However, the relationship between the content of these groups and the humidity sensing capability of GO-based sensors remains unclear. In the present work, we investigate the role of oxygen-containing functional groups in the humidity sensing performance by oxidizing graphite with mesh numbers 80–120, 325, and 8000 using the Hummers method, resulting in GO-80, GO-325, and GO-8000. Infrared spectroscopy (IR) and X-ray photoelectron spectroscopy (XPS) were used to identify the types and quantification of oxygen-containing functional groups. Molecular dynamics simulation is used to simulate the adsorption energy, intercalation dynamics, and hydrogen bonding of water molecules. Electrochemical tests were used to compare the adsorption/desorption time and response sensitivity of graphene oxide to humidity. It is proposed that hydroxyl and carboxyl groups are the main contributing groups to humidity sensing. GO-8000 shows a relatively fast response time, but the large number of carboxyl groups will hinder intercalation of water molecules, thus exhibiting lower sensitivity. This research provides a reference for the future development of graphene-based sensors, catalysts, and environmental materials.

## 1. Introduction

As a key material for the next generation of artificial skin and flexible electronics, it should be able to sense changes in the microenvironment (temperature, humidity) quickly and accurately [1,2,3,4]. Graphene is widely used in humidity sensing due to its large specific surface area (2620 m^2^/g) and high electron transmission rate (10^6^ m/s) [5,6,7]. In order to improve its processing performance, the Hummers method is often used to peel off graphite to obtain well-dispersed graphite in the aqueous phase [8]. The resulting oxygen-containing functional groups include but are not limited to carboxyl groups (–COOH) and ketone groups (=O), hydroxyl (–OH) and ether bonds (–O–), giving graphene its polarity and hydrophilicity. The presence of oxygen-containing functional groups can change the electronic structure of graphene, while the adsorption of water molecules may affect its conductive properties, which is crucial for the design of graphene-based electronic devices [9,10,11,12].

In the field of catalysis, graphene is often used as an active center support for different molecules, metal catalysts, or as an active center to participate in the catalytic cycle directly [13]. The relationship between external diffusion and reaction pressure drop caused by graphite size is of important relevance in the field of catalytic reactions and water treatment membranes. In composite materials, oxygen-containing functional groups can promote the interaction between graphene and other materials and improve the dispersion and interface stability of composite materials [14]. Tripathi et al. stacked molybdenum sulfide on graphite layers, achieving superior performance through synergies between two-dimensional materials [15]. In energy and environmental applications, proton conduction mediated by water molecules on the surface of graphene oxide provides a theoretical basis for the development of new hydrolysis and fuel cells. Reaction molecular dynamics studies have shown that both the epoxy groups and hydroxyl groups on the surface of graphene oxide can effectively adsorb water molecules to form a hydrogen bond network [16]. 

It is generally believed that oxygen-containing functional groups are mainly at the edges of graphene when it is oxidized [17]. Then, once the edge adsorption sites are occupied by a large number of water molecules, will they prevent more water molecules from entering the interlayer? Moreover, excessive oxygen content in graphene oxide may lead to a reduction in electrical conductivity, which can affect the sensor’s performance. Thus, it is a delicate balance to maintain optimal oxygen content to provide both good conductivity and high sensitivity for humidity sensing. However, it should be noted that the correlation between the type and content of oxygen on the humidity sensing sensitivity is not clear yet. Since different preparation methods, experimental conditions, and graphite sources all affect the structure of graphene oxide, its precise structure has been difficult to determine. Therefore, it is necessary to conduct experimental and theoretical quantification of the content of functional groups of graphene oxide. The graphite reported in the literature is generally 200 mesh, and the corresponding average size is about 75 microns. The packed bed reactor used in industrial catalysis is generally 80 mesh, while the 8000-mesh size can be used in the field of nanomaterials. Therefore, three representative graphites of 80, 325, and 8000 mesh were selected to study the size effect.

Moreover, to investigate the interaction of graphene oxide and water molecules, researchers often combine experiments and theoretical calculations for analysis. For example, density functional theory (DFT) calculations can be used to explore the interaction at atomic and molecular levels, as well as the impact of this interaction on electron transport, thereby further explaining the mechanism of humidity sensing [18,19,20]. To investigate the interaction of graphene oxide and water molecules, we have obtained a series of graphene oxide films and achieved effective control of film thickness uniformity through pulling and spin coating methods [21,22]. Previous studies have shown that water molecules can be distributed near graphene oxide sheets and undergo ultrafast adsorption and desorption. This is determined by the polarity, size, and electron-withdrawing ability of the intercalated molecules. The optical response time is on the order of hundreds of milliseconds, which is far lower than semiconductor impedance humidity sensing materials [23]. A sensor based on TiO_2_/KNbO_3_ achieved a response time of 3 s and a relatively long recovery time of 163 s [24].

**Scheme 1:**
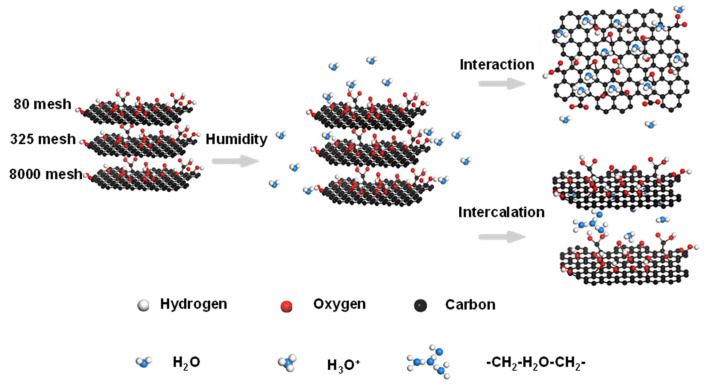
Schematic diagram of GO synthesis and humidity sensing mechanism.

Herein, the modified Hummers method was used to exfoliate graphite and then the obtained GO aqueous solution was spin-coated to prepare GO films. Combined with molecular dynamics simulations, the performance of the type and number of oxygen-containing functional groups in response to humidity was discussed. The research results are of great significance for clarifying the dynamics and sites of intercalation of water molecules by oxygen-containing functional groups, and providing experimental and theoretical foundations for the future development of humidity-responsive materials. Research shows that as the mesh number increases from 80 to 325 to 8000, the degree of oxidation and the relative content of oxygen-containing functional groups increase. Under 15% RH (relative humidity) humidity conditions, the interlayer spacing of GO sheets expands from 9.01 Å to 9.57 Å. Under medium- and high-humidity conditions, GO-8000 has the best comprehensive humidity sensing performance (Scheme 1).

## 2. Results and Discussion

### 2.1. Basic Characterization of GOs

The D band and G band are prominent features in the Raman spectra of graphite and graphene materials. The D band is typically located around 1350 cm⁻¹. This band is associated with the breathing modes of sp^2^ atoms in rings and is indicative of defects or disorder in the graphite lattice. The G band is found around 1580 cm⁻¹. This band arises from the bond stretching of all pairs of sp^2^ atoms in both rings and chains and is a characteristic feature of graphitic materials. 

**Figure 1:**
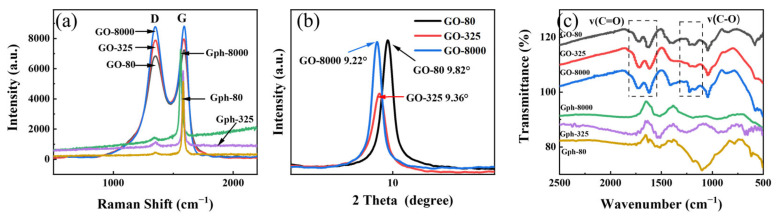
(**a**) Raman spectra of the raw graphite Gph-80, Gph-325, and Gph-8000 and oxidized graphene GO-80, GO-325, GO-8000; (**b**) XRD of GO-80, GO-325, and GO-8000; and (**c**) FTIR of GO-80, GO-325, GO-8000, Gph-80, Gph-325, and Gph-8000.

As shown in Figure 1a, these bands are used to assess the oxidation degree and structure of carbon-based materials, with the intensity of the D band relative to the G band providing information about the level of disorder or defects present. Due to crystal symmetry, the D band peak at 1350 cm^−1^ was quite weak in the original graphite (Gph). The Raman signal intensity indicates the degree of bond cleavage of the graphene oxide lattice and the influence of the edge structure. The intensity ratio I_D_/I_G_ of the two characteristic peaks of GO-80, GO-325, and GO-8000 is 0.8739, 0.9430, and 0.9606, respectively, indicating that the defect density of the three samples increases as the sheet size decreases. GO-8000 shows the highest defect density, which suggests that under the same oxidation conditions, the 8000-mesh graphite has a smaller C–C plane lattice than other graphite during the oxidation process, when the degree of C=C bond cleavage and edge defects is higher, and oxygen-containing functional groups are more likely to be formed.

In the XRD diffraction spectrum (Figure 1b), there is no (002) diffraction peak at 26.5°, indicating that the graphite is completely oxidized. The characteristic peak positions of (001) of the three GO samples with different mesh sizes are 9.82°, 9.36°, and 9.22°, respectively. The corresponding interlayer spacings are calculated to be 9.01Å, 9.42Å, and 9.57Å, respectively, indicating that the larger the sheet, the smaller the GO layer spacing. The broad peak band at 1627 cm^−1^ in the infrared spectrum of the GO was attributed to the skeleton vibration of the benzene ring in unoxidized graphene. The broad absorption peak band around 3400 cm^−1^ is the stretching vibration of O–H. The absorption vibration peaks generated by oxygen-containing functional groups at 1398 cm^−1^ and 1731 cm^−1^ (Figure 1c) originated from the deformation of –C–O–H and the stretching of –C=O in the –COOH group, respectively. Compared with the vibration peak of the benzene ring, GO-8000 has a higher C=O vibration intensity, which is consistent with the above-mentioned Raman and XRD tests. The absorption vibration peak at 1050 cm^−1^ is the vibration of –C–O functional group. The surface of the prepared GO material has abundant oxygen-containing functional groups, and it is expected to show a more sensitive effect on the interaction between the material and water molecules.

**Figure 2:**
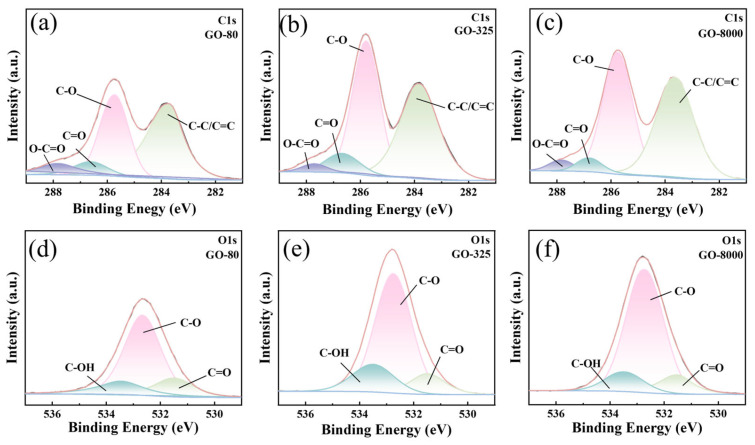
(**a**–**c**) Both experiment result (line) and peak fitting results (colored area) of XPS C1s and (**d**–**f**) XPS O1s of GO-80, GO-325, and GO-8000.

XPS is used to identify the existence form and content of carbon and oxygen elements in the GOs. The C1s spectra of GO-80, GO-325, and GO-8000 corresponding to the binding energies of –C–C/–C=C–, –C–O, –C=O, and –O–C=O were found at around 283.8 eV, 285.8 eV, 286.7 eV, and 287.8 eV, respectively (as shown in Figure 2a–c). The O1s peak centers at 531.5 eV, 532.8 eV, and 533.0 eV were attributed to C–OH, –C–O, and –C=O, respectively (as shown in Figure 2d–f). The peak shift in the O1s peak position of GO with different meshes is due to the conductivity of GOs. After performing deconvolution fit of XPS peaks, the O1s spectra are obtained (as shown in Figure 2d–f). Integration of the peaks suggested that most O atoms in the three samples existed in the form of –C–O. In the C1s spectra, the –O–C=O content increased from 1.21% to 3.05%, –C=O increased from 2.64% to 6.12%, and the proportion of –C–O increased from 47.25% to 54.06% as the GO size decreased. In the O1s spectra, the ratio of –C=O increased from 6.22% to 10.11%, the proportion of –C–OH increased from 0.69 to 7.18%, and the proportion of –C–O decreased from 93.08% to 82.71%, as summarized in Table 1.

As the mesh number increases, the relative content of oxygen element in the sample increases. Among them, the epoxy group located on the GO base plane accounts for the main component, and the carbonyl and carboxyl groups located on the edge account for a smaller proportion. Under the same oxidation conditions, GO with a smaller sheet has more oxygen-containing functional groups.

**Figure 3:**
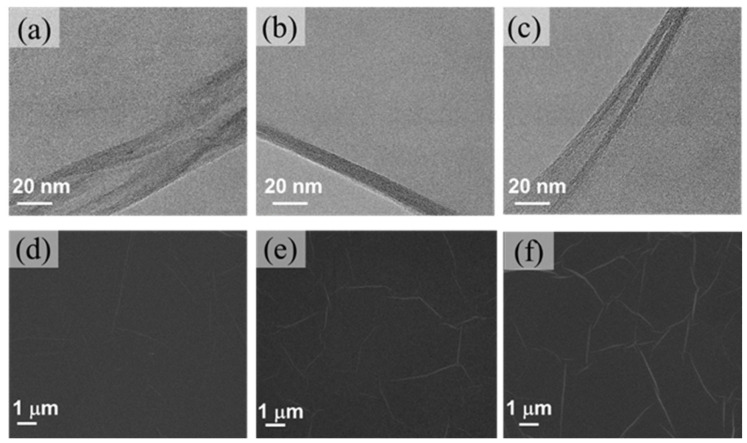
(**a**–**c**) TEM images of f GO-80, GO-325, and GO-8000; (**d**–**f**) SEM images of GO-80, GO-325, and GO-8000.

Graphene oxide is often used in the form of thin films for humidity sensing, so it is necessary to observe the morphology of the thin films. It can be clearly observed from the TEM images (Figure 3a–c) that the graphite is well exfoliated. The GO dispersion dropped onto the silicon wafer is relatively smooth overall, except for some wrinkles (Figure 3d–f). The wrinkles on the surface of the films are the essential characteristics of the stable two-dimensional graphene oxides. During the synthesis process of GO, the oxidation of –C=C– bonds lead to the destruction of the conjugated structure and promotes the generation of wrinkles. This phenomenon can simultaneously increase the surface area and enhance the contact efficiency with other molecules, but it will reduce the conductivity of GOs.

### 2.2. Effect of Humidity on Layer Spacing

**Figure 4:**
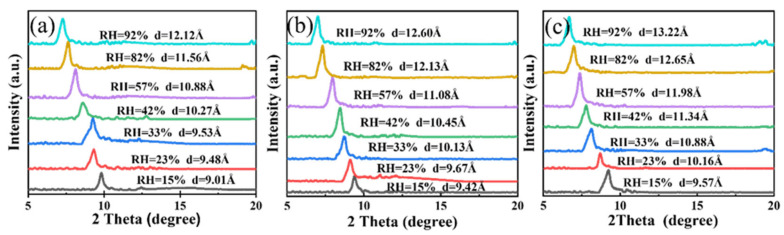
(**a**) Layer spacing of GO-80, (**b**) GO-325 and (**c**) GO-8000 under 92%, 82%, 57%, 42%, 33%, 23% and 15% RH.

The degree of oxidation of graphene oxide nanosheets has a significant impact on the microstructure of GO membranes. As the relative humidity increases from 15% to 92%, the interlayer spacing expands from 9.01 Å, 9.42 Å, and 9.57 Å to 12.12 Å, 12.60 Å, and 13.22 Å, respectively (as shown in Figure 4). The interlayer spacing distribution is affected by the oxidation degree of graphene and the amount of intercalated water. It is the sp^3^ hybrid structure and oxygen-containing functional groups in GO that adsorb water molecules; however, as the interlayer spacing continuously increases, there is a balance point where a certain amount of water is adsorbed due to the osmotic pressure of water in between layers. Moreover, due to the existence of the sp^2^ hybrid conjugated structure, the rigidity of GO nanosheets is improved and the further expansion of the interlayer spacing is limited. Therefore, GOs with more oxygen-containing functional groups tend to have more obvious performance in changes in layer spacing.

### 2.3. Molecular Dynamics Simulation

In order to clearly simulate the adsorption amount, adsorption energy, and number of interacting hydrogen bonds of water molecules in the three samples, a molecular dynamics simulation system was constructed based on the C and O element contents and the relative content data of each functional group calculated in XPS. The GO multi-layer structure was constructed based on the XRD layer spacing. OPLS-AA (the all-atom optimized potentials for liquid simulations) force field was used to describe H_2_O molecules, and a large number of water molecules were added to the system and given random movement speeds. For the adsorption process and interaction of water molecules, the NPT (isothermal–isobaric ensemble) integrated system with a step size of 1.0 fs was used to optimize the simulation system. The pressure was set to 1 atm, the temperature was 298.15 K, and a Nose–Hoover controlled thermostat was used. Newton’s equations were used to explain the atomic motion process and the v-Verlet algorithm was used to solve it. LAMMPS (Large-scale Atomic/Molecular Massively Parallel Simulator, 23 Jun 2022-Update 4) software was used to complete the dynamics simulation process.

**Figure 5:**
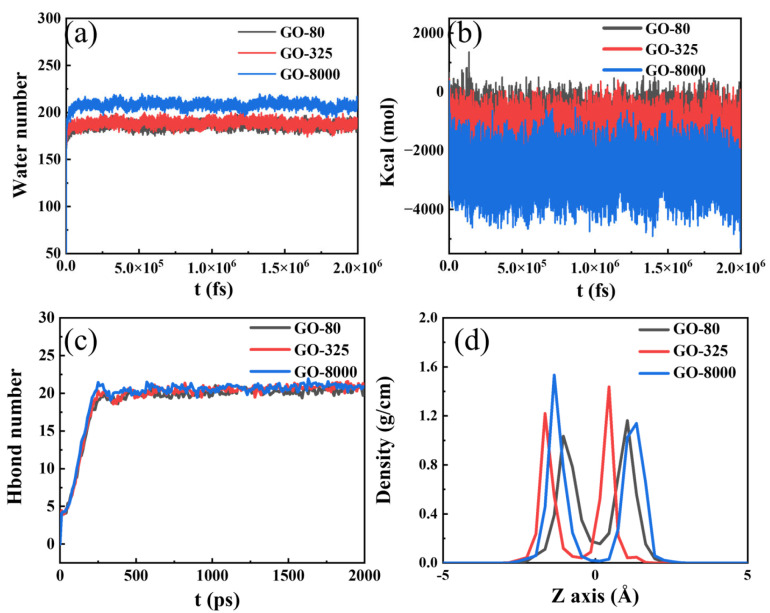
Molecular dynamics simulation of GO-80, GO-325, and GO-8000. (**a**) The amount of adsorbed water over time, (**b**) the absorption energy and (**c**) the variation in hydrogen bonds between adsorbed water molecules and oxygen-containing functional groups on GO surface over time, (**d**) the density of adsorbed water around the GO layer.

When the amount of adsorbed water molecules reaches stability, the adsorption capacity of GO-8000 is 10.71% more than that of GO-80 and 9.53% more than that of GO-325 (Figure 5a). The adsorption energy between water molecules and oxygen-containing functional groups is a key aspect of intermolecular interactions. This interaction is mainly formed through hydrogen bonds, and the strength and stability of hydrogen bonds directly affect the ability of water molecules to combine with oxygen-containing functional groups. The absorption energy of water molecules on the surface of GOs is shown in Figure 5b. The higher the negative value of these data, the stronger the interaction. Then, the order of interaction strength is GO-8000 > GO-80 > GO-325 accordingly. The results were further confirmed by the number of hydrogen bonds, as shown in Figure 5c. Due to the difference in the relative content of oxygen-containing functional groups in the prepared materials, the most hydrogen bonds were found in GO-8000, followed by GO-325, and GO-80 had the least hydrogen bonds.

The adsorbed water molecules are distributed closely to the functional groups, showing a higher peak relative to the center of the slit. Moreover, this part of the water molecules overlaps with the functional groups at the plane, indicating that a small number of water molecules fill the gaps between the functional groups, and the part of water molecules distributed close to the plane is greatly affected by the plane and the functional groups. The distribution characteristics of water molecules in different slits have certain regularity. The peak heights of the water molecule peaks in the slit system are 0.9~1.1 g/cm^3^, 1.0~1.2 g/cm^3^, and 1.1~1.3 g/cm^3^ (Figure 5d), respectively. The arrangement of water molecules in the slit suggested characteristics different from those of bulk water molecules. Planes and functional groups have a greater influence on the water molecules in the slit.

### 2.4. Humidity Response Performance

Graphene oxide itself is not a typically *p*-type or *n*-type material. In its natural state, graphene oxide may exhibit a certain *p*-type behavior. This is because the oxygen-containing functional groups on the surface can serve as electron acceptors and extract electrons from the graphene plane, leaving more holes, resulting in *p*-type carrier-dominated conductivity. The migration of electrons and holes has an important impact on humidity sensing. 

**Figure 6:**
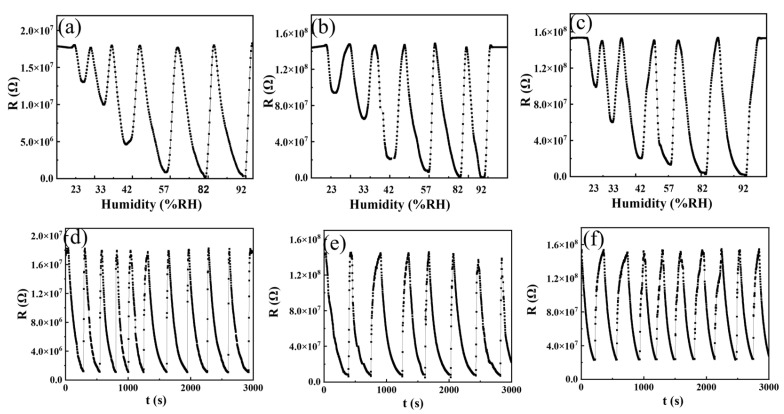
(**a**–**c**) The resistance curves of GO-80, GO-325, and GO-8000 at 15~92% RH; (**d**–**f**) GO-80, GO-325, and GO-8000 sensing performance switching between 15% and 92% RH humidity.

The three sample dispersions were drop-coated onto interdigitated electrodes, and a micro-voltage was applied to test the humidity sensing performance at 15~92% RH (Figure 6a–c). As the humidity increases, the resistance decreases until a certain humidity condition is reached. The adsorbed water molecules can accept electrons from GO to form hydrated electrons. This electron transfer will cause changes in the electronic structure of GO, thereby affecting its conductivity. In addition to the movement of electrons, the adsorption of water molecules may also lead to the generation of holes. In GO, the presence of water molecules can prompt oxygen atoms to release electrons to adjacent carbon atoms, leaving holes at the oxygen atoms. Such an increase in holes will also change the conductivity of GO. Due to the migration of electrons and holes, the conductivity of GO changes with changes in humidity. Under low-humidity conditions, the conductivity of GO is low because there are not enough free electrons and the conductive path is clear. Under high-humidity conditions, the conductivity of GO will increase due to the occupation of electrons by water molecules and the increase in the number of holes.

The electrochemical stability of the three electrodes was tested by switching RH between 15% and 92% (Figure 6d–f). GO-80 showed the smallest resistance, the slowest response speed, and the fastest recovery time during the test process. The GO-8000 sample had the largest resistance value, the fastest response speed, and the slowest recovery speed. The response times and recovery times of the three samples are 6 s, 3 s, and 2 s and 12 s, 28 s, and 48 s, respectively. The desorption time of water molecules on the sample film is much longer than its adsorption time. This is because, during the process from adsorption to complete desorption of water molecules, the molecules need to obtain enough energy to overcome the attraction between adjacent molecules and thus be able to detach from the sample. For water molecules that enter between the GO sheets and undergo hydration reactions, the water molecules easily form larger clusters due to hydrogen bonds, and the desorption process often requires more energy and time. The desorption process lags behind the adsorption process and the adsorption plots are consistent with the type IV isotherm, indicating single-layer and multi-layer adsorption on micro-mesoporous adsorption materials. As the humidity increases, the sample resistance continues to decrease, and the curve becomes flat after reaching a certain humidity condition. 

**Figure 7:**
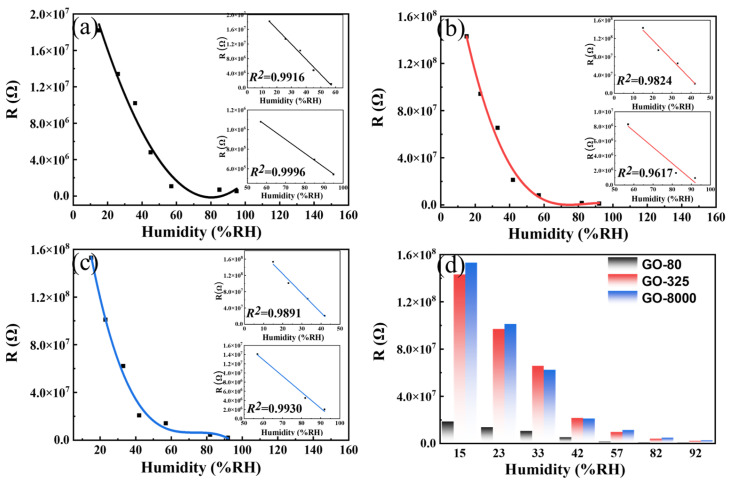
(**a**–**c**) The fitting curves of the response trend of GO-80, GO-325, and GO-8000 at 15~92% RH humidity (the squares represent experimental data and the line is fitting curve); (**d**) the corresponding resistance values of the samples under different humidity conditions.

The corresponding maximum resistances of the three samples are 1.82 × 10^7^ Ω, 1.43 × 10^8^ Ω, and 1.53 × 10^8^ Ω, respectively. When the ambient humidity reaches 92%, the corresponding resistance values of the samples are 5.41 × 10^5^ Ω, 1.87 × 10^6^ Ω, and 2.32 × 10^6^ Ω, respectively (Figure 7a–c). Linear fitting was performed on the humidity response sensitivity changes of the resistance of the samples in the humidity ranges of 15% to 57% RH and 57 to 92% RH. It was found that when the humidity reached about 57%, the decline curves of the resistance values of the three samples all slowed down significantly (Figure 7d).

**Figure 8:**
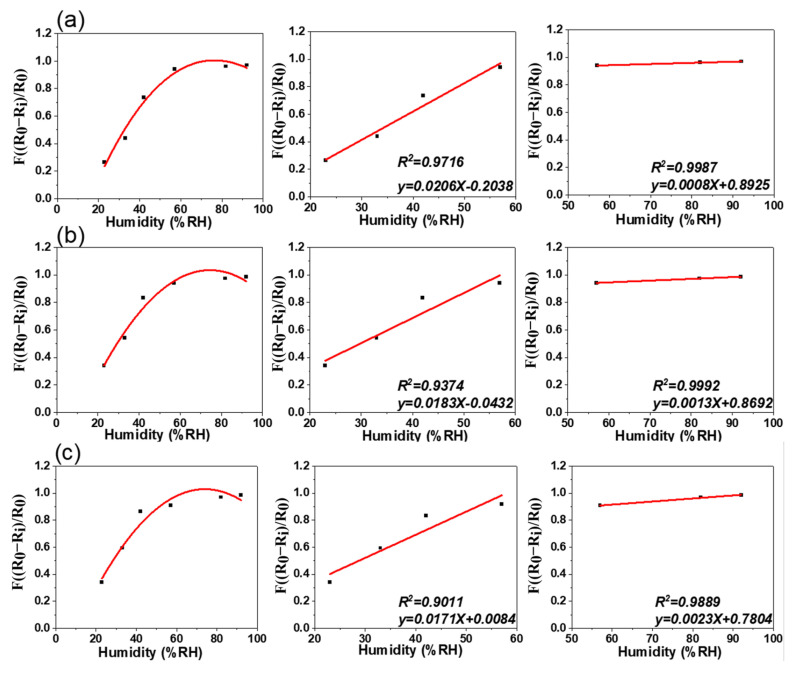
(**a**–**c**) The sensitivity fitting curves of the humidity response of GO-80, GO-325, and GO-8000 at 15% to 92% RH and sensitivity linear fit of the humidity response of 15 to 57% and 57% to 92% RH, respectively (the dots represent experimental data, and the line is fitting curve).

The illustration of sensitivity change curves of GO-80, GO-325, and GO-8000 in response to humidity is shown in Figure 8. Linear fitting was performed for the humidity response sensitivities of 15~57% RH and 57~92% RH, respectively.

$$R_{P}=\frac{R_{0}-R_{i}}{R_{0}}$$

*R*_0_ is the resistance of the sample under initial conditions (15% RH, room temperature), *R_i_* is the resistance under the corresponding humidity, and ΔRH is the change in humidity relative to the initial conditions. *R_p_* is the sample response sensitivity.

When the humidity changes from 15% to 23%, the corresponding sensitivities of the samples are 26.37%, 32.38, and 33.99%, respectively. The humidity response sensitivity shows an obvious gradient phenomenon, and as the humidity increases, the response sensitivity of GO-8000 is higher than that of GO-325 and GO-80 in the RH range of 15% to 57%. When the humidity reaches 92%, GO-8000 shows lower response sensitivity than GO-325 and GO-80. At this time, the corresponding sensitivities of the three samples are, respectively, 97.03%, 98.69%, and 98.48%.

### 2.5. Humidity Response Mechanism

The introduction of oxygen-containing functional groups into graphene materials has different effects on their conductivity. The hydroxyl groups in GO-8000 can form hydrogen bonds with the surface of graphene, thereby hindering charge transport and thus reducing the conductivity of the material to a certain extent. The most hydrogen bonds were generated in GO-8000 (Figure 5c). The presence of carboxyl groups has a more significant impact on the conductivity of GO. The XPS results of both C1s and O1s show that GO-8000 has a large number of carboxyl groups. Carboxyl groups can interact with the graphene surface and change its electronic structure. In addition, the dissociation of carboxyl groups may lead to ion transport, further reducing the conductivity (Figure 7d). Epoxy groups can form covalent bonds with the graphene surface, destroying the conjugated structure of graphene, thereby affecting its conductivity, such as GO-80 samples.

At low humidity (RH < 15%), water molecules enter GO through diffusion and interact with surface oxygen-containing functional groups and tend to be between GO sheets, and GO is in a low-hydration state with less H^+^ and free electrons. There are many ion barriers, which hinder the movement of ions and have high resistance. When the humidity gradually increases, the role of hydroxyl and carboxyl groups is to increase the occupation of electrons by water molecules and increase the number of holes, and the mechanism of water adsorption begins to change. The adsorbed water molecules that have diffused into the inside of the sheets quickly reduce the drag by increasing the hydronium ions between the GO sheets. After RH > 57%, the concentration of water molecules reaches a critical value. Limited by the number of oxygen-containing functional groups, the number of hydrated ions reaches the upper limit. At this time, the resistance reduction mainly depends on the change in the content of adsorbed water in the layer. Moreover, when the adsorbed water content reaches a certain level, water aggregates will form within the sheet, hindering subsequent water adsorption and slowing down the resistance reduction rate (Figure 7b,c). Therefore, the large number of carboxyl groups in GO-8000 will hinder further intercalation of water molecules and exhibit lower sensitivity (Figure 8).

## 3. Materials and Methods

### 3.1. Materials

Graphite powder of 80–120 mesh (≥99.95%), 325 mesh (≥99.95%), and 8000 mesh (≥99.95%) was purchased from Macklin Biochemical Technology Co., Ltd., Shanghai, China. Potassium permanganate (98%), phosphorus pentoxide (98%), sulfuric acid (98%), hydrochloric acid (AR), hydrogen peroxide (98%), and potassium persulfate (98%) were ordered from Sinopharm Chemical Reagents Co., Ltd., Shanghai, China.

### 3.2. Instruments

Infrared (IR) spectra were measured using an IR Nicolet 10 FT-IR spectrometer (Thermo Fisher, Waltham, MA, USA). Hitachi Regulus 8220 (Hitachi High-Technologies Corporation, Tokyo, Japan) scanning electron microscopy (SEM) was used to examine the surface and cross-section of films. The accelerating voltage was set to 5 kV. The samples were first sputtered with gold for 20 s prior to SEM analysis. Gas sensing tests were performed by CGS–MT (Beijing Sinoya Pavilion, Beijing, China). The method is calibrated by gas chromatography and the accuracy of the gas monitor is less than ±5% full scope (F.S) of the gas monitor. X-ray photoelectron spectrometer (Thermo Fisher ESCALAB Xi Thermo Fisher, Waltham, MA, USA) was used to analyze and quantize the existing form of oxygen and carbon atoms.

### 3.3. Synthesis of the GO

First, we weighed 0.5000 g of graphite powder of different mesh sizes, 0.8500 g P_2_O_5_, and 0.8500 g K_2_S_2_O_8_ into a 50.00 mL round-bottomed flask, and 4.00 mL concentrated H_2_SO_4_ was added dropwise into it. This mixture was allowed to react at 80 °C for 4.5 h. After that, the product was suction filtered and washed with deionized water until pH = 6.0~7.0 to obtain a dry filter cake. The above pre-oxidized graphite was put into a round-bottomed flask and 20.00 mL concentrated H_2_SO_4_ was added. A total of 2.5000 g of KMnO_4_ was added in multiple batches within half an hour under 0 °C ice bath conditions. The reaction was carried out for 30 min and then transferred to a 35 °C water bath. The reaction was continued at this temperature for 2 h before 41.50 mL of deionized water was added dropwise. After 2 h, 116.50 mL of deionized water was added, and 3.30 mL of H_2_O_2_ was added dropwise and the reaction was continued for 2 h. After the reaction was completed, we washed the product three times with 5.00% HCl hydrochloric acid solution to remove residual H_2_SO_4_ and other unreacted substances in the solution. Then, deionized water was used to wash the product until pH = 6.0.

### 3.4. Preparation of Interdigitated Electrodes

Diluted graphene oxide (GO) solutions were prepared by reducing the concentration of the three acquired GO solutions to 1 mg/mL. Then, these solutions were carefully dispensed onto the interdigitated electrode plates, ensuring uniform coverage across the entire electrode surface. The thickness of each film is 200 ± 10 nm measured by SEM. The samples were allowed to dry in air at room temperature. Subsequently, the electrical resistance of the samples over a relative humidity range from 15% to 92% was measured.

## 4. Conclusions

In summary, graphene oxides with different density of the oxygen-containing functional groups (–COOH, –OH, –C–O–C–, –C=O) were produced by oxidizing graphite through the modified Hummers method. During the oxidation process, the spacing between GO sheets is significantly expanded due to the generation of oxygen-containing functional groups, destruction of the crystal lattice, formation of edge defects, and its wrinkle characteristics. The generation of hydrophilicity and the expansion of interlayer spacing significantly improve the electrochemical performance of GO in humid environments. GO-80 with a lighter degree of oxidation showed the fastest desorption time among the three samples, and GO-8000, with the highest degree of oxidation, showed the highest resistance, fastest response speed, and best water adsorption performance. This experimental result is consistent with its structural characteristics.

As the relative humidity of the environment increases, all three GO films show the characteristic of increasing interlayer spacing, and exhibit a threshold consistent with the degree of oxidation under each humidity condition. Due to the difference in the number of oxygen-containing functional groups, –OH has the greatest impact on sample performance during the humidity response process, followed by –COOH. Molecular dynamics results show that the adsorbed water molecules are distributed close to the functional groups. The mechanism shows that the water molecules enter between the sheets, enhancing ion conduction, and expand the interlayer spacing. When a certain humidity is reached, limited by the number of oxygen-containing functional groups, the number of hydrated ions reaches the upper limit. At this time, the resistance decrease mainly depends on the change in the content of adsorbed water in the layer.

## Figures and Tables

**Table 1:** The form and relative content of C1s and O1s.

Sample	C1s					O1s			
	O–C=O	C=O	C–O	C–C/C=C	C–Total (%)	C–OH	C–O	C=O	O–Total (%)
GO-80	1.21	2.64	47.45	48.70	67.91	0.69	93.08	6.22	32.09
GO-325	1.90	3.50	50.22	44.38	67.68	0.96	91.56	7.49	32.32
GO-8000	3.05	6.12	54.06	36.77	67.45	7.18	82.71	10.11	32.55

## Data Availability

Data will be made available on request.
